# Classic Hodgkin Lymphoma Refractory for ABVD Treatment Is Characterized by Pathologically Activated Signal Transduction Pathways as Revealed by Proteomic Profiling

**DOI:** 10.3390/cancers14010247

**Published:** 2022-01-04

**Authors:** Bent Honoré, Maja Dam Andersen, Diani Wilken, Peter Kamper, Francesco d’Amore, Stephen Hamilton-Dutoit, Maja Ludvigsen

**Affiliations:** 1 Department of Biomedicine, Aarhus University, 8000 Aarhus, Denmark; bh@biomed.au.dk (B.H.); wilken0210@gmail.com (D.W.); 2 Department of Haematology, Aarhus University Hospital, 8200 Aarhus, Denmark; majaaner@rm.dk (M.D.A.); Petekamp@rm.dk (P.K.); frandamo@rm.dk (F.d.); 3 Department of Clinical Medicine, Aarhus University, 8000 Aarhus, Denmark; stephami@rm.dk; 4 Department of Pathology, Aarhus University Hospital, 8200 Aarhus, Denmark

**Keywords:** Hodgkin lymphoma, proteomics, prognosis, treatment

## Abstract

**Simple Summary:**

Classic Hodgkin lymphoma (cHL) patients refractory to standard ABVD chemo-therapy are known to have a dismal prognosis. This has led to the hypothesis that ABVD treatment-sensitive and ABVD treatment-refractory tumours are biologically distinct. In this study, cHL patients refractory to standard ABVD treatment show subtle but significant differences in protein expression that enable clustering of the two response groups, thus indicating differences between ABVD sensitive and refractory patients at the molecular level, and thereby strengthening the hypothesis that ABVD sensitive and ABVD refractory tumours may be biologically distinct.

**Abstract:**

In classic Hodgkin lymphoma (cHL), the tumour microenvironment (TME) is of major pathological relevance. The paucity of neoplastic cells makes it important to study the entire TME when searching for prognostic biomarkers. Cure rates in cHL have improved markedly over the last several decades, but patients with primary refractory disease still show inferior survival. We performed a proteomic comparison of pretreatment tumour tissue from ABVD treatment-refractory versus ABVD treatment-sensitive cHL patients, in order to identify biological differences correlating with treatment outcome. Formalin-fixed paraffin-embedded tumour tissues from 36 patients with cHL, 15 with treatment-refractory disease, and 21 with treatment-sensitive disease, were processed for proteomic investigation. Label-free quantification nano liquid chromatography tandem mass spectrometry was performed on the tissues. A total of 3920 proteins were detected and quantified between the refractory and sensitive groups. This comparison revealed several subtle but significant differences in protein expression which could identify subcluster characteristics of the refractory group. Bioinformatic analysis of the biological differences indicated that a number of pathologically activated signal transduction pathways are disturbed in ABVD treatment-refractory cHL.

## 1. Introduction

Classic Hodgkin lymphoma (cHL) accounts for 10–15% of all lymphomas. This B-cell-derived malignant lymphoproliferative disease is diagnosed in approximately 2–3 persons per 100,000/year [1]. Although the incidence has been fairly constant over the last 30 years, lymphoma-related mortality has reduced considerably. The age-specific incidence shows a bimodal distribution with peaks at 20–30 years and 70–75 years [2]. The standard treatment regimen for limited stage cHL is ABVD (adriamycin, bleomycin, vinblastine, dacarbazine) with involved node radiation therapy. There is no international consensus on the treatment of advanced stage cHL. Therapy is either ABVD or BEACOPP (bleomycin, etoposide, adriamycin, cyclophosphamide, vincristine, procarbazine, and prednisolone), depending on patient age, performance score, comorbidities, and the traditions of the treating institution. The more intensive BEACOPP regimen has a higher response rate and a progression-free survival over 80%, compared with 65% in ABVD-treated patients [2]. The higher response rate is at the cost of substantial early morbidity (e.g., life threatening infections, infertility) and risk of late mortality due to secondary malignancies (e.g., secondary leukaemia and myelodysplasia) [3,4].

Newly diagnosed young cHL patients have a cure rate > 80%, but approximately 5–10% of cHL patients are refractory to initial treatment, and another 10–30% of patients will experience a relapse after achieving a first complete remission [2,5]. Patients with primary refractory disease have a dismal prognosis and long-term disease-free survival after second-line chemotherapy produces low response rates (<10% of patients) [6].

The tumour microenvironment (TME) in cHL is characterized by a minority population of neoplastic Hodgkin and Reed–Sternberg (HRS) cells, embedded in a heterogeneous background infiltrate of non-neoplastic bystander cells, including B and T cells, but also eosinophils, basophils, macrophages, plasma cells, and mast cells, as well as vascular and other stromal cells [7,8,9,10,11,12]. Studies have shown that HRS cells interact with the surrounding microenvironment by production of chemokines and through direct cell–cell contact [13,14]. The scarcity of neoplastic cells make the entire TME relevant when looking for prognostic biomarkers.

Immunohistochemistry, flow and mass cytometry, and gene expression profiling techniques have provided a better understanding of the TME in cHL, and have contributed to new treatment approaches, e.g., anti-CD30 antibody and programmed cell death protein 1 inhibitors (PD-1 inhibitors). In order to better predict treatment response, we and others have previously performed proteomic-based studies on fresh frozen tissue samples from cHL patients [15,16,17]. However, advances in the proteomic profiling technology have enabled the use of formalin-fixed paraffin-embedded (FFPE) tumour-tissue samples. This allows analyses to be performed on a much greater number of tumour specimens, avoiding the limitations imposed by the requirement for fresh frozen archival tissue from patients with long follow up. Little is known about factors predisposing to primary refractory cHL. Although the International Prognostic Score (IPS) and interim-PET/CT-scan are used in risk stratification and treatment guidance, no unequivocal predictive pre-therapeutic markers for primary refractory cHL have so far been identified.

In this study, we investigated the protein expression in tumours from cHL patients using nano high-pressure liquid chromatography tandem mass spectrometry (nanoLC-MS/MS), according to ABVD treatment outcome, in order to identify putative protein biomarkers of primary refractory disease.

## 2. Materials and Methods

### 2.1. Patients

All patients diagnosed with cHL at the Department of Haematology, Aarhus University Hospital in the period of 2001–2015 were identified through the Danish Pathology Registry [18] and the Danish National Lymphoma Registry (LYFO) [19,20]. All tumour biopsies were reviewed and reclassified by an experienced haematopathologist according to the 2017 WHO Classification of Tumours of the Haematopoietic and Lymphoid Tissues to validate the cHL diagnosis [1]. Patients were included based on first-line treatment schedules with ABVD or ABVD-like treatment (ABVD/COPP (cyclophosphamide, oncovin, procarbazine, prednisone)), with or without additional consolidating radiotherapy. Patients were included in the study if sufficient pre-therapeutic FFPE tumour tissue was available from an excision biopsy. Data on demographics, clinicopathological features and treatment outcome were acquired by crosslinking with the LYFO registry and, if needed for data completeness, with patient records (Table 1). Patients were grouped according to treatment outcome, i.e., primary treatment-refractory cHL patients (*n* = 15) versus treatment-sensitive cHL patients (*n* = 21). Primary refractory disease was defined as patients with progressive disease during treatment or relapse within 3 months after the end of treatment. Sensitive disease was defined as patients that following treatment, achieved a complete remission (CR) and showed no sign of progression or relapse with a minimum follow-up period of 3 years from diagnosis.

### 2.2. Samples/Sample Preparation

Pretherapeutic tumour biopsies from patients with adequate clinical information and available FFPE archive tissue samples were included in the study. FFPE tissue samples were collected, processed, and stored using standard procedures. Preparation of tissue for proteomics was performed essentially as previously described [21]. In short, seven 10-micron paraffin sections were cut and paraffin was removed with xylene. The tissue was then incubated with ethanol followed by repeated incubations in decreasing ethanol/water, and finally dried. The rehydrated tissue was lysed in 5% sodium deoxycholate (SDC), 20 mM triethylammonium bicarbonate (TEAB) and then incubated at 99 °C for 1 h and slowly chilled to room temperature. After homogenization and sonication, protein concentration was estimated with infrared spectrometry, and up to 100 μg was used for trypsin digestion in 0.5% SDC and 20 mM TEAB at 37 °C overnight, using a filter technique (Microcon Centrifugal Filter Devices 30 K from Merck KGaA, Darmstadt, Germany). SDC was removed using phase extraction with ethyl acetate and acidification with trifluoroacetic acid. Digests were dried by vacuum centrifugation resuspended in 100 mM TEAB, and peptide concentration was estimated by fluorescence. Samples were dried and resuspended to a concentration of 1 μg/μL and 2 μg were used for label-free quantification (LFQ) nLC-MS/MS.

### 2.3. Label-Free Nano Liquid Chromatography—Tandem Mass Spectrometry (LFQ nLC-MS/MS)

LFQ nLC-MS/MS was run essentially as described [22] using a μ-Precolumn (300 μm × 5 mm, C18 PepMap100, 5 μm, 100 Å, Thermo Fisher Scientific Instruments, Waltham, MA, USA) for trapping peptides and an analytical column (EASY-Spray Column, 750 mm × 75 μm, PepMap RSCL, C18, 2 mm, 100 Å, Thermo Fisher Scientific Instruments, Waltham, MA, USA) for separation of peptides with a nano liquid chromatography system (Ultimate 3000 Dionex, Thermo Fisher Scientific Instruments, Waltham, MA, USA). Peptides were eluted with a 4 h gradient and analysed on an Orbitrap Fusion Tribrid mass spectrometer (Thermo Fisher Scientific Instruments, Waltham, MA, USA). The universal method settings were used [22]. Fluoranthene was used as an internal mass calibrant by activating the EASY-IC.

### 2.4. Data and Bioinformatic Analyses

The 36 raw data files were used to search the Homo sapiens reviewed database from Uniprot, downloaded on 10th August 2019 by MaxQuant (v1.6.6.0) [23] with the settings for label-free quantification (LFQ) as previously described [22]. Perseus (v1.6.6.0) [24] was used for subsequent analysis, where the data were filtered by removal of proteins only identified by site, identified in the reverse database, and those that were potential contaminants. Data were log_2_ transformed and proteins were required to be identified by at least 2 unique peptides. This resulted in the identification of a total set of 3920 proteins, combined in all 36 patient tissues. For some analyses, the data were filtered, so proteins with missing values were filtered out. This resulted in 1197 proteins present in all samples. For other analyses, especially the bioinformatic analyses, the biological information is higher if data is not filtered too hard. For some analyses, the data were filtered so that the proteins were present in at least 60% of the samples. This resulted in 2473 proteins for analyses. Imputation of data to fill missing values was not used. Using these filtrations, bioinformatic analysis was performed through the use of ingenuity pathway analysis (IPA) (Qiagen Inc., Hilden, Germany, https://www.qiagenbioinformatics.com/products/ingenuity-pathway-analysis (accessed on 8 September 2021)). The algorithms developed for use in IPA are described by Krämer et al. [25]. The observed significant differences in expression levels were subtle, with fold changes between 1.18 and 1.58 (Supplementary Table S1).

### 2.5. Statistical Analysis

Distribution differences of clinical characteristics between groups were analysed using Fisher’s exact test. All *p*-values were 2-sided and values were regarded statistically significant if *p* < 0.05. All statistical analysis were performed using STATA software version 17.0 (StataCorp. 2021. Stata Statistical Software: Release 17. College Station, TX, USA: StataCorp LLC.).

## 3. Results

### 3.1. Study Cohort

Protein expression in pretherapeutic FFPE tumour samples from cHL patients were assessed by LFQ nLC-MS/MS and compared according to ABVD treatment outcome, i.e., primary treatment-refractory cHL patients (*n* = 15) versus treatment-sensitive cHL patients (*n* = 21). Demographics and clinicopathological features of the cHL patients are listed in Table 1. The entire cohort included 15 (41.7%) females and 21 (58.3%) males with a median age of 36 years (range 15–79). With regard to sex, IPS, histology, B-symptoms and Ann Arbor stage, no statistically significant differences were observed between the two groups. Bulky disease was more prevalent in the refractory group (67%), compared with the ABVD-sensitive group (48%) (*p* = 0.028).

### 3.2. Protein Expression

**Figure 1:**
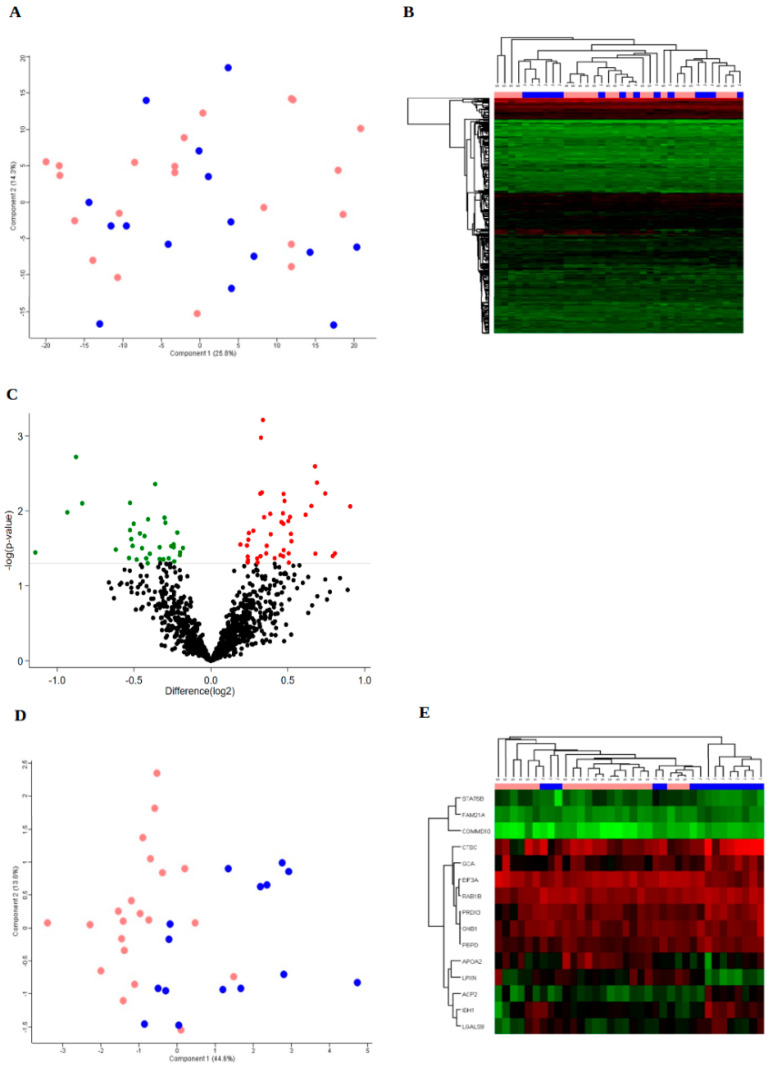
Differentially expressed proteins based on 1197 proteins detected in all patient samples. Principal component analysis (**A**) and unsupervised clustering (**B**) based on 1197 proteins detected in all patient samples. Pink = s, sensitive; Blue = r, refractory. (**C**) Volcano plot based on the 1197 proteins expressed in all patient tumours comparing the treatment-refractory versus the treatment-sensitive groups (log2 values). The grey horizontal line marks the threshold of *p* < 0.05 of 79 significantly differentially expressed proteins. Fifteen proteins were differentially expressed at *p* < 0.01 and 1 protein at *p* < 0.001. Red: Upregulated. Green: Downregulated. (**D**) Principal Component Analysis, and (**E**) unsupervised clustering based on 15 differentially expressed proteins (*p* < 0.01) in all patient samples. Pink = s, sensitive; Blue = r, refractory.

Protein expression in tumour samples from all patients (*n* = 36) were analysed and resulted in a total of 3920 protein groups that were detected with at least two unique peptides (Supplementary Table S2). Various numbers of missing values were obtained in each sample and the removal of the proteins that were not detected in all samples left 1197 proteins that were detected in each of the 36 samples (Supplementary Table S3). By using the expression levels of these proteins as the basis for a principal component analysis (PCA), no separation of the response groups was found (Figure 1A). In addition, unsupervised hierarchical clustering showed no clear clustering of the patients (Figure 1B). Comparison between the treatment-refractory and the treatment-sensitive groups revealed a set of differentially expressed proteins, 79 proteins at *p* < 0.05, 15 proteins at *p* < 0.01 and 1 protein at *p* < 0.001, Figure 1C. PCA and unsupervised hierarchical clustering based on the 15 differentially expressed proteins (*p* < 0.01) separated a cluster consisting of 8 of the 15 treatment-refractory patients (Figure 1D–E).

**Figure 2:**
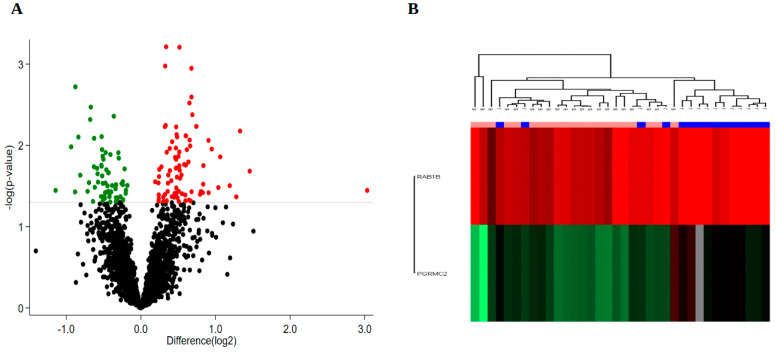
Differentially expressed proteins detected in at least 60% of the analysed samples. (**A**) Volcano plot of 2473 proteins found in at least 60% of patient samples. The grey horizontal line marks the threshold of *p* < 0.05 of 145 significantly differentially expressed proteins. Twenty-six proteins were differentially expressed at *p* < 0.01 and 2 proteins at *p* < 0.001. Red: Upregulated. Green: Downregulated. (**B**) Unsupervised clustering based on expression of 2 proteins (*p* < 0.001). Pink = s, sensitive; Blue = r, refractory.

To extract as much information as possible, we expanded the number of proteins identified to include proteins that were identified in at least 60% of the analysed samples. This procedure was performed in each of the treatment groups in order to reveal which biological differences could be observed between the two groups. This method detected 2473 proteins (Supplementary Table S4). By comparing the two treatment groups using these proteins, we obtained differentially expressed proteins amounting to 145 proteins at *p* < 0.05, 26 proteins at *p* < 0.01 and 2 proteins at *p* < 0.001, Figure 2A. Interestingly, the expression level of the 2 proteins detected at the significance level *p* < 0.001, Ras-related protein Rab-1B (*RAB1B*) and membrane-associated progesterone receptor component 2 (*PGRMC2*), could separate a cluster of 11 patients of the treatment-refractory group (Figure 2B), which included the 8 patients of the cluster shown in Figure 1D–E. This indicates that there are biological differences between the refractory and the sensitive groups that are at least partially important for the response to treatment.

### 3.3. Bioinformatics

**Figure 3:**
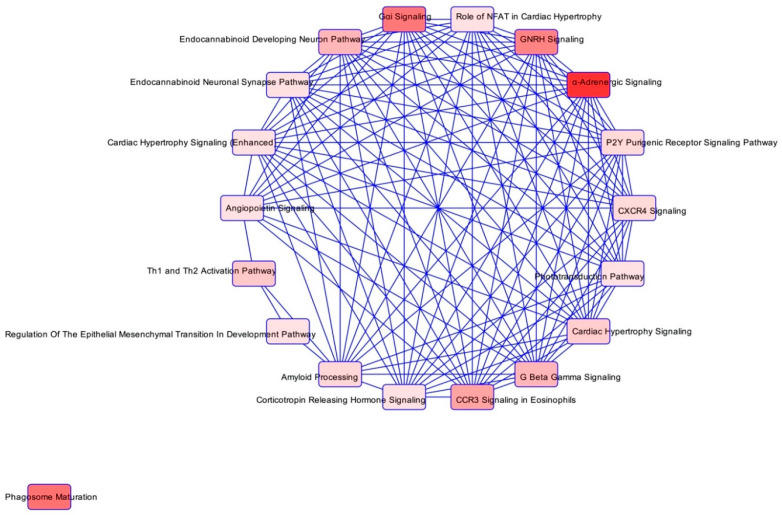
Ingenuity pathway analysis in ABVD refractory and ABVD sensitive patients. Overlapping of the pathways found to be enriched by ingenuity pathway analysis based on 1197 proteins identified. The vast majority of pathways belong to signalling pathways. The proteins involved are listed in Table 2.

In order to reveal the biological differences between the sensitive and the refractory treatment groups, we used IPA to find enriched pathways. By using the 79 differentially expressed proteins significantly changed among the 1197 detected proteins in all samples analysed, we found signal transduction pathways to be especially enriched (Table 2). The vast majority of the pathways were overlapping, only one pathway being an orphan pathway, not sharing any proteins with the other 18 pathways identified (Figure 3). Two of the pathways had z-scores at ≥2 (namely ‘Gαi signalling’ and ‘*CXCR4* signalling’), indicating that they were not only enriched in the analysis, but that these pathways were also significantly activated. In the ‘*CXCR4* signalling’ pathway, four proteins were all discretely upregulated in the therapy resistant group (*GNAI2*, *GNB1*, *RALB* and *PAK2*). The first three of these proteins are shared with the ‘Gαi signalling’ pathway, that also included downregulation of *PRKACB* in the therapy resistant group. By expanding the IPA analysis to include the 2473 proteins identified in at least 60% of samples in each group, a similar tendency was apparent, i.e., several signal transduction pathways were enriched (Supplementary Figure S1). Two additional proteins in the ‘*CXCR4* signalling’ pathway were detected, *CRK* and *GNAQ*, both upregulated (Supplementary Table S1). This analysis also identified two pathways that were significantly activated (z-scores ≥ 2), the ‘Ephrin receptor signalling’ and ‘Endocannabinoid neuronal synapse’ pathways both sharing proteins with the previously mentioned pathways, in addition to the upregulated protein *ARPC5* (Supplementary Table S1).

**Figure 4:**
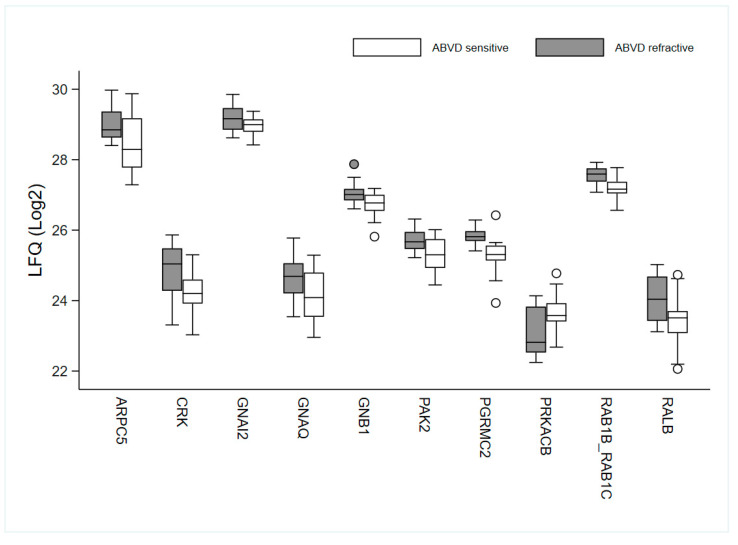
Box plot of the levels of ten selected proteins, *APRC5*, *CRK*, *GNAI2*, *GNAQ*, *GNB1*, *RALB*, *RAB1B*, *PAK2*, *PGRMC2*, and *PRKACB* in the ABVD sensitive and refractory group. Label-free quantification (LFQ).

Thus, IPA revealed a number of proteins participating in signal transduction pathways that show a very discrete upregulation in the therapy resistant group (*GNAI2*, *GNB1*, *RALB*, *PAK2*, *CRK*, *GNAQ*, *ARPC5*) and one downregulated protein (*PRKACB*). These proteins, together with the two proteins, *RAB1B* and *PGRMC2*, that define the sub-cluster of 11 resistant patients, might be interesting candidates as prognostic biomarkers or as potential targets for therapy, among other proteins participating in the pathways. The levels of these proteins in the ABVD sensitive and refractory groups are shown in Figure 4.

## 4. Discussion

This is the first study to report the use of MS-based proteomics with FFPE material to correlate protein expression profiles in cHL tumour tissues with treatment response and clinical outcome. In our cohort, bulky disease was more prevalent in the ABVD refractory group and jointly, the refractory group tended towards more adverse clinicopathological features at diagnosis, as is consistent with, but not exclusive to, cHL patients in high risk of refractory disease. Still, identification of a putative predictive marker of those patients in high risk of refractory disease remains an unmet clinical need. In the present study, the protein expression differences seen between the cHL patient with either ABVD refractory or sensitive treatment responses were discrete. The large majority of protein fold changes were below 2, indicating that the biological differences between the groups are subtle. Nevertheless, in spite of the subtle changes detected, bioinformatic analysis revealed enrichment of proteins participating in signal transduction pathways. Especially two pathways, ‘Gαi signalling’ and ‘*CXCR4* signalling’, were not only enriched, but were also found to be activated in the therapy resistant group. Several of the proteins participating in these signal transduction pathways were slightly upregulated in the therapy resistant group. Taken together, the analyses identified a number of proteins (e.g., *GNAI2*, *GNB1*, *RALB*, *PAK2*, *CRK*, *GNAQ*, and *ARPC5*) with fold changes between 1.18 and 1.58. In addition, the expression level of two proteins (*RAB1B* and *PGRMC2*), could separate a subcluster consisting of 11 of the 15 patients in the therapy resistant group. These two proteins exhibited fold changes of 1.26 and 1.43. Thus, they may, together with other proteins participating in these signal transduction pathways, be prime candidate targets for future treatment strategies.

In a top-down proteomic study based on 2D-PAGE analysis conducted on freshly frozen tissues from 14 patients, we previously identified galectin-1 as being upregulated in tumours from a therapy resistant subgroup [17]. In the present analysis based on 36 patients, we found a similar tendency for galectin-1 upregulation in the therapy resistant group, although the observed change was nonsignificant (fold change 1.33, *p* = 0.193).

*CXCR4* is a chemokine G protein coupled receptor. The sole known ligand to *CXCR4* is *CXCL12* [26]. *CXCR4* is involved in a number of physiological and pathological processes. *CXCR4*, once activated by *CXCL12*, activates Gαi, that downstream activates the Ras/Raf/MEK/ERK pathway, thereby modulating progressions in the cell cycle [27]. In parallel, *CXCR4* can activate the phosphatidyl-inositol 3 kinase (PI3K)–RACα serine/threonine protein kinase (AKT) pathway [28]. Our data show both enriched and activated ‘Gαi signalling’ and ‘*CXCR4* signalling’ in the refractory cHL group. In view of this, it is interesting that the AKT deregulation and constitutive activation in HRS cells has previously been demonstrated as a key feature in the cHL pathogenesis [29].

*‘CXCR4* signalling’ also plays a role in chemotherapy resistance and *CXCR4* inhibition can possibly act as a chemotherapy sensitizer [30,31]. *CXCR4* antibodies can disrupt adhesive tumour/stroma interactions and mobilize cancer cells from their protective stromal microenvironment, making them more accessible to chemotherapeutic agents. *CXCR4* signalling modulators, e.g., plerixafor, an anti-*CXCR4* antibody, is being tested in clinical studies in both solid and haematologic cancers (e.g., acute myeloid leukaemia (AML), chronic lymphatic leukaemia (CLL) and multiple myeloma (MM) [26,32,33]. Currently, plerixafor is being used for mobilization of haematopoietic stem cells in patients with MM and lymphoma. Other anti-*CXCR4*-based treatments are on the horizon, including, LY2624587, another anti-*CXCR4* monoclonal antibody, which caused dose-dependent apoptosis in vitro and in vivo in human haematologic cancer cells and provided a significant survival benefit in a disseminated lymphoma model [34]. Our study demonstrated upregulation of the *CXCR4* signalling pathway in ABVD refractory cHL cases, indicating HL as a possible target for anti-*CXCR4* antibody treatment.

The endocannabinoid neuronal synapse pathway comprises two G-protein coupled receptors (GPCR), and cannabinoid receptors CB1 (*CNR1*) and CB2 (*CNR2*). Ligand activation of the GPCR results in conformational changes in the receptor, which is transmitted through the associated *Gα* subunit to initiate intracellular signalling cascades [35,36]. *CNR1* is widely expressed in the CNS, but is also found in other tissues, whereas *CNR2* has a more localized distribution, being predominantly expressed in the tissues and cells of the immune system [37,38]. The pathways stimulated by cannabinoids are involved in cell-to-cell signalling and interaction, and have important roles in cell growth and differentiation [39]. In cancer, the endocannabinoid system seems to play a dual role in tumorigenesis and metastatic spread. Cannabinoids might exert their antitumour effects by several different mechanisms, including induction of apoptosis, cell cycle arrest, and inhibition of angiogenesis and metastasis [40]. Aberrant expression of components of the endocannabinoid system, especially *CNR1* and *CNR2*, seems to be related to tumour growth and progression. Some cannabinoids, probably at low concentrations, may increase tumour proliferation and induction of tumour growth [38]. The relationship to cancer prognosis and disease outcome is dependent on the cancer type. In pancreatic, colon, and prostate carcinomas, an altered endocannabinoid system is a marker of invasiveness and of poor cancer prognosis [38]. In breast cancer studies, conflicting results have been published [38]. In diffuse large B-cell lymphoma (DLBCL), high serum levels of endocannabinoid 2-arachidonoylglycerol has been reported to play a role in the pathogenesis or progression of the disease [41]. In Hodgkin lymphoma, abundant *CB1R* was found in CD30+ HRS cells of cHL, whereas the surrounding reactive, non-neoplastic cell infiltrate was largely *CNR1*-negative. The same study reported an antiapoptotic role for *CNR1* in cHL and hypothesized that CB receptors function as a survival factor for HRS cells and concluded that endocannabinoid system activation promotes tumour cell growth [42]. Further studies of the role of the endocannabinoid system in cHL are needed, but so far, data suggests that targeting CB receptors may have therapeutic potential for the treatment of lymphoma.

In addition to the aforementioned pathways, ephrin receptor signalling was also upregulated in our study. Erythropoietin-producing hepatoma (Eph) receptor signalling consists of receptor tyrosine kinases that bind to membrane-anchored ligands, ephrins that influence cell behaviour attraction/repulsion, adhesion/de-adhesion in axon guidance, cell migration, angiogenesis and synaptic plasticity, and thus affect tumour growth, angiogenesis, and metastasis in cancer [43,44,45,46]. In Eph receptor signalling, both oncogenic and tumour suppressive roles have been described [46]. Studies have, for example, demonstrated that *EPHA2* can act both as a tumour promoter and a tumour suppressor, depending on its phosphorylation status [46,47]. In glioblastoma and prostate cancer cell lines, overexpression of *EPHA2* resulted in oncogenic signals. In non-small cell lung cancer and breast cancer, overexpression of *EPHA2* correlated to tumorigenesis and risk of metastasis [44]. *EPHA3* and *EPHA4* reduced the development of metastasis and could possibly be useful biomarkers for prognosis in lung cancer [48]. In follicular lymphoma (FL), the *EPHA7* receptor has been identified as a tumour suppressor [49]. The same group detected inactivation deletions in 72% of FL samples. Currently, the role of the Eph signalling pathway in cHL has not been investigated; however, anti-ephrin receptor antibodies are being tested in clinical settings in other cancers, including haematologic malignancies, and this may be an interesting novel approach in refractory cHL patients [48,50].

In summary, the signalling pathways identified in our study have previously been demonstrated to be disturbed in cancers, including haematologic malignancies. Especially, the *CXCR4* signalling is currently being explored in AML, CLL, and MM [26,32,33]. In addition, our data contain new information on activated pathways in cHL, representing possible novel participants in the pathogenesis of cHL. Thus, it seems that the biological differences between the ABVD refractory and sensitive groups of cHL patients may be associated with perturbations in these signalling pathways. In particular, the tumours in the refractory patient group show changes in distinct pathways that were significantly activated as compared with tumours from the sensitive group. Any proteomic technique has shortcomings and only detects a fraction of the proteome. Therefore, it should be mentioned that several other proteins participating in these pathways also could be warranted to investigate. Future studies focusing on the proteins detected here as well as other proteins participating in these pathways are thereby warranted. In addition, correlations to other parameters including EBV status and PD1/PDL1 could be analysed. Further validation studies are necessary to establish the relevance of these pathways in the clinical setting of cHL.

## 5. Conclusions

Tumours from ABVD refractory cHL patients show changes in distinct pathways that were significantly activated as compared with tumours from ABVD-sensitive cHL patients, thus indicating differences in the tumours at the molecular level.

## Figures and Tables

**Table 1:** Baseline clinicopathological characteristics of the study cohort.

Characteristics	All Patients ( *n* = 36)		Refractory Group ( *n* = 15)		Sensitive Group ( *n* = 21)	
	No. of Patients	%	No. of Patients	%	No. of Patients	%
**Median age years** (range)	36 (15–79)		32 (15–72)		36 (17–79)	
**Sex** (*n* = 36)						
Male	21	58.3	8	53.3	13	61.9
Female	15	41.7	7	46.7	8	38.1
**IPS** (*n* = 35)						
0–3	29	82.9	10	71.4	19	90.5
>3	6	17.1	4	28.6	2	9.5
**Histology** (*n* = 36)						
NS	31	86.1	12	80.0	19	90.5
MC	4	11.1	2	13.3	2	9.5
LD	1	2.8	1	6.7	0	0
**B-Symptoms** (*n* = 36)						
No	16	44.4	5	33.3	11	52.4
Yes	20	55.6	10	66.7	10	47.6
**Ann Arbor** (*n* = 36)						
I–II	20	55.6	6	40.0	14	66.7
III–IV	16	44.4	9	60.0	7	33.3
**Bulk** (*n* = 34)						
No	21	61.8	5	38.5	16	76.2
Yes	13	38.2	8	61.5	5	23.8
**Treatment modality** (*n* = 36)						
ABVD	29	80.6	13	86.7	16	76.2
ABVD/COPP	7	19.4	2	13.3	5	23.8
**CMT** (*n* = 21)						
ABVD+RT	16	76.2	7	100.0	9	64.3
ABVD/COPP+RT	5	23.8	0	0	5	35.7

Abbreviation: IPS: international prognostic score; NS: nodular sclerosis; MC: mixed cellularity; LD: lymphocyte-depleted; ABVD: doxorubicin, bleomycin, vinblastine, dacarbazine; COPP: cyclophosphamide, oncovin, procarbazine, prednisone; CMT: combined modality treatment; RT: radiotherapy.

**Table 2:** Canonical pathways based on 1197 proteins identified in all samples.

Ingenuity Canonical Pathways	−log(*p*-Value)	Ratio	z-Score	Molecules
α-Adrenergic Signalling	2.56	0.312		*GNAI2*, *GNB1*, *PRKACB*, *PYGB*, *RALB*
Phagosome Maturation	1.99	0.184		*ATP6V1G1*, *CTSC*, *CTSZ*, *DYNLT1*, *NAPA*, *RAB5C*, *RAB7A*
Gαi Signalling	1.97	0.286	2	*GNAI2*, *GNB1*, *PRKACB*, *RALB*
GNRH Signalling	1.92	0.227	1.342	*GNAI2*, *GNB1*, *PAK2*, *PRKACB*, *RALB*
CCR3 Signalling in Eosinophils	1.75	0.25		*GNAI2*, *GNB1*, *PAK2*, *RALB*
G Beta Gamma Signalling	1.66	0.235	1	*GNAI2*, *GNB1*, *PRKACB*, *RALB*
Endocannabinoid Developing Neuron Pathway	1.66	0.235		*GNAI2*, *GNB1*, *PRKACB*, *RALB*
Th1 and Th2 Activation Pathway	1.57	0.222		*CHD4*, *LGALS9*, *NCSTN*, *STAT5B*
Cardiac Hypertrophy Signalling	1.54	0.185	0.447	*GNAI2*, *GNB1*, *HSPB1*, *PRKACB*, *RALB*
Amyloid Processing	1.5	0.273		*CAPN2*, *NCSTN*, *PRKACB*
*CXCR4* Signalling	1.49	0.211	2	*GNAI2*, *GNB1*, *PAK2*, *RALB*
P2Y Purigenic Receptor Signalling Pathway	1.49	0.211	0	*GNAI2*, *GNB1*, *PRKACB*, *RALB*
Phototransduction Pathway	1.42	0.4		*GNB1*, *PRKACB*
Regulation of the Epithelial Mesenchymal Transition in Development Pathway	1.42	0.4		*NCSTN*, *S100A4*
Role of NFAT in Cardiac Hypertrophy	1.42	0.2	0	*GNAI2*, *GNB1*, *PRKACB*, *RALB*
Cardiac Hypertrophy Signalling (Enhanced)	1.41	0.154	0.816	*DIAPH1*, *GNAI2*, *GNB1*, *HSPB1*, *PRKACB*, *RALB*
Angiopoietin Signalling	1.4	0.25		*PAK2*, *RALB*, *STAT5B*
Endocannabinoid Neuronal Synapse Pathway	1.4	0.25		*GNAI2*, *GNB1*, *PRKACB*
Corticotropin Releasing Hormone Signalling	1.31	0.231		*ARPC5*, *GNAI2*, *PRKACB*

## Data Availability

The datasets analysed during the current study are available upon reasonable request.

## Supplementary Materials

The following are available online at www.mdpi.com/article/10.3390/cancers14010247/s1, Supplementary Table S1: Canonical pathways based on 2473 proteins identified, Supplementary Table S2: 3920 proteins identified combined in all samples, Supplementary Table S3: 1197 proteins identified in all samples, Supplementary Table S4: 2473 proteins identified in at least 60% of samples in each group, Supplementary Figure S1: Overlapping of the pathways found to be enriched by ingenuity pathway analysis (IPA) based on 2473 proteins identified in at least 60% of patient samples in each group.
