# Supplementary figures and images for: Classic Hodgkin Lymphoma Refractory for ABVD Treatment Is Characterized by Pathologically Activated Signal Transduction Pathways as Revealed by Proteomic Profiling
Source: Cancers (Basel). 2022 Jan 4;14(1):247. doi: 10.3390/cancers14010247 (PMC8750842; doi:10.3390/cancers14010247)

Supplementary Figure S1

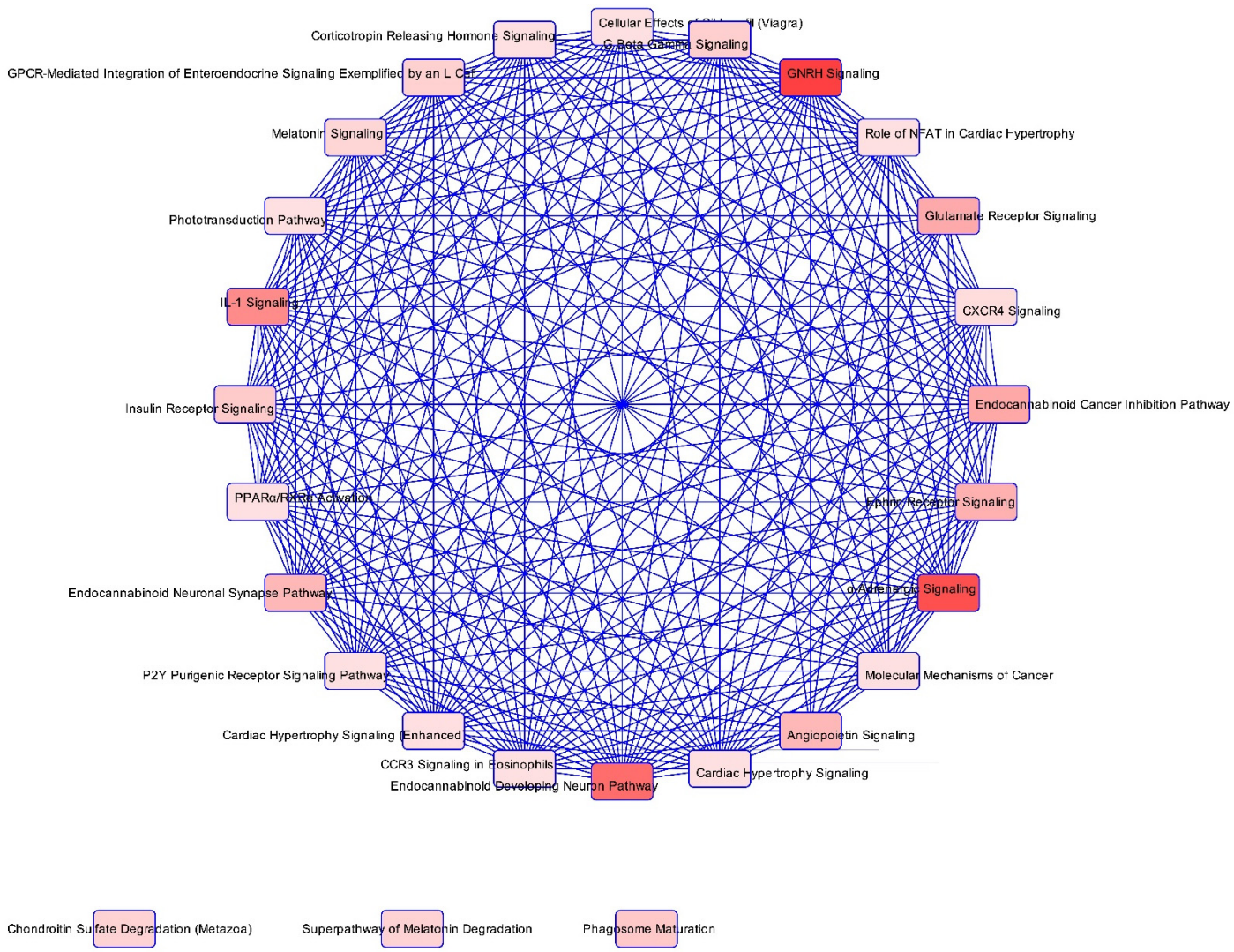

Supplement: Supplementary file 1 [file cancers-14-00247-s001.zip › Supplementary Figure S1.pdf]
